# A Neuro-ontology for the neurological examination

**DOI:** 10.1186/s12911-020-1066-7

**Published:** 2020-03-04

**Authors:** Daniel B. Hier, Steven U. Brint

**Affiliations:** Department of Neurology and Rehabilitation, University of Illinois at Chicago, 912 S. Wood Street (MC 796), Chicago, IL 60612 USA

**Keywords:** UMLS Metathesaurus, Ontology, Neurological examination, Electronic health records, SNOMED CT

## Abstract

**Background:**

The use of clinical data in electronic health records for machine-learning or data analytics depends on the conversion of free text into machine-readable codes. We have examined the feasibility of capturing the neurological examination as machine-readable codes based on UMLS Metathesaurus concepts.

**Methods:**

We created a target ontology for capturing the neurological examination using 1100 concepts from the UMLS Metathesaurus. We created a dataset of 2386 test-phrases based on 419 published neurological cases. We then mapped the test-phrases to the target ontology.

**Results:**

We were able to map all of the 2386 test-phrases to 601 unique UMLS concepts. A neurological examination ontology with 1100 concepts has sufficient breadth and depth of coverage to encode all of the neurologic concepts derived from the 419 test cases. Using only pre-coordinated concepts, component ontologies of the UMLS, such as HPO, SNOMED CT, and OMIM, do not have adequate depth and breadth of coverage to encode the complexity of the neurological examination.

**Conclusion:**

An ontology based on a subset of UMLS has sufficient breadth and depth of coverage to convert deficits from the neurological examination into machine-readable codes using pre-coordinated concepts. The use of a small subset of UMLS concepts for a neurological examination ontology offers the advantage of improved manageability as well as the opportunity to curate the hierarchy and subsumption relationships.

## Background

The aggregation of clinical data for big data projects from electronic health records poses challenges [1–5]. Much of the clinical data in electronic health records (EHRs) are represented as free text. Although progress is being made in the conversion of free text into structured data by natural language processing (NLP), these methods are not in general use [6–10]. The entry of data about neurological patients in EHRs into large databases requires a method for converting symptoms (patient complaints) and signs (examination abnormalities) into machine-readable codes.

$$ \mathbf{Signs}+\mathbf{Symptoms}=\mathbf{Findings}\rightarrow \mathbf{Concepts}\rightarrow \mathbf{Machine}\ \mathbf{Codes} $$

In this paper, we will use *findings* to mean *signs and symptoms collectively*. This conversion process can be facilitated by an ontology that links neurological signs and symptoms to appropriate concepts and machine-readable codes.

### The neurological examination and history

There is no standard neurological examination and history [11–13]. Depending on the examiner and patient, a neurological examination and history may take as little as three minutes or greater than 30 min to complete. Furthermore, there is no generally accepted format for recording the neurological examination and history. Some neurologists use an outline, some use a table, and others use a narrative. No agreed-upon terminology exists for recording the neurological examination and history. During an examination, a neurologist elicits symptoms such as weakness, slowness, memory loss, speech impairment, blurred vision, numbness, tingling, pain, or imbalance and abnormalities in the mental status, cranial nerves, motor system, sensory system, reflexes, coordination, and gait. Due to a lack of standard terminology, identical neurological abnormalities may be described variously. For example, a failure to abduct the eye may be variously recorded as a sixth nerve palsy, an abducens palsy, or an abducens nerve weakness. Similarly, an upgoing great toe upon stimulation of the plantar surface of the foot may be recorded variously as Babinski sign, upgoing toe, positive Babinski, or upgoing plantar response.

### The need for an ontology for the neurological examination

If neurological findings are to be converted into machine-readable codes, an ontology is needed [14]. At a minimum, an ontology for the neurological examination and history that supports big data applications should do the following:

1. Neurological findings with the same meaning but different wording should be represented by the same concept.
2. A unique machine-readable code should be assigned to each concept**.**
3. The ontology should be organized hierarchically with a root concept and should support subsumption (**is_a** relationships).
4. The ontology should have a *scope* (breadth of coverage) and a *granularity* (depth of coverage) that enables it to capture findings recorded during a neurological examination faithfully.
5. Synonymous terms should be linked to each concept.

Some ontologies have other useful characteristics. Many ontologies attach a definition to each concept. Other ontologies, SNOMED CT in particular, allow simple concepts to be combined to form more complex concepts. For example, the concept |ankle reflex| can be combined with |absent| and is equivalent to |absent ankle reflex|. This is known as post-coordination [15]. Concept ontologies that are organized hierarchically support the calculation of inter-concept distances [16–24].

### The UMLS Metathesaurus

The UMLS Metathesaurus is not an ontology per se [25, 26]. The 2019 AB release is a curated compendium of 155 distinct terminology sources with 4,258,810 concepts. Each concept is linked to a unique machine-readable code and a specified name. For example, the concept aphasia has the CUI (concept unique identifier) **C0003537**. Each CUI is eight characters in length and starts with the letter C, followed by seven digits. The concept *aphasia* is contributed 58 times to UMLS from 58 different source terminologies. Each contribution of *aphasia* appears in the UMLS with the same CUI but a different atom unique identifier (AUI). The UMLS maintains concept synonyms as *normalized concept names.* The 2019AB release has 11,882,429 normalized concept names, each with a unique LUI. For example, the concept *aphasia* has 22 English language synonyms in UMLS, including |loss of words|, |losing words|, |loss of power of expression or comprehension|, |aphasic syndrome|, and |difficulty finding words|. The UMLS Metathesaurus also maintains files with concept definitions, files with relationships between concepts (child-parent, etc.), and files with ontology hierarchies (paths from each concept to the root concept).

## Methods

### Test-phrases

We abstracted the neurological examinations from 419 published neurological case studies [27–33]. Based on the neurological findings, we created a dataset of 2386 test-phrases for encoding as concepts by an ontology. Normal findings were ignored.

### Mapping of test-phrases to UMLS concepts

Test phrases were mapped to UMLS concepts using the UMLS browser. Except for a few concepts, we ignored laterality (e.g., right ataxia, left ataxia, and bilateral ataxia were all mapped to the UMLS concept ataxia |**C0004134**|. We mapped the 2386 test phrases to 601 concepts. Concepts recurred a mean of 3.9 ± 5.5 times (range 1 to 47 times) in the 419 test teaching cases. The 15 most common neurology concepts are shown in Table 1.

**Table 1:** Fifteen most common test concepts found in neurological teaching cases^a^

Concept	CUI	Count	Proportion (%)
hyperreflexia	C0151889	47	2.0
headache	C0018681	45	1.9
unstable gait	C0231686	41	1.7
bilateral extensor plantar responses	C0422917	38	1.6
dysarthria	C0013362	32	1.3
confused	C0009676	31	1.3
hearing impaired	C1384666	31	1.3
appendicular ataxia	C0750937	27	1.1
hemiparesis	C0018989	24	1.0
papilledema	C0030353	23	1.0
diplopia	C0012569	22	0.9
neck stiffness	C0151315	22	0.9
impaired memory	C0233794	22	0.9
ataxic gait	C0751837	20	0.8
disorientation	C0233407	19	0.8

^a^CUI is from UMLS Metathesaurus browser. Proportion based on 2386 total concepts abstracted from 419 published neurology teaching cases

### Construction of NEO (neurological examination ontology)

We reviewed the neurological history and examination as presented in three standard textbooks [11–13] and identified 1100 findings, which were either signs or symptoms. Each of the 1100 concepts was entered into the Protégé ontology editor [34] and consisted of the following:

- Concept name
- UMLS CUI (concept identifier)
- SNOMED CT SCTID (concept identifier) if available
- Parent concept

**Fig. 1:**
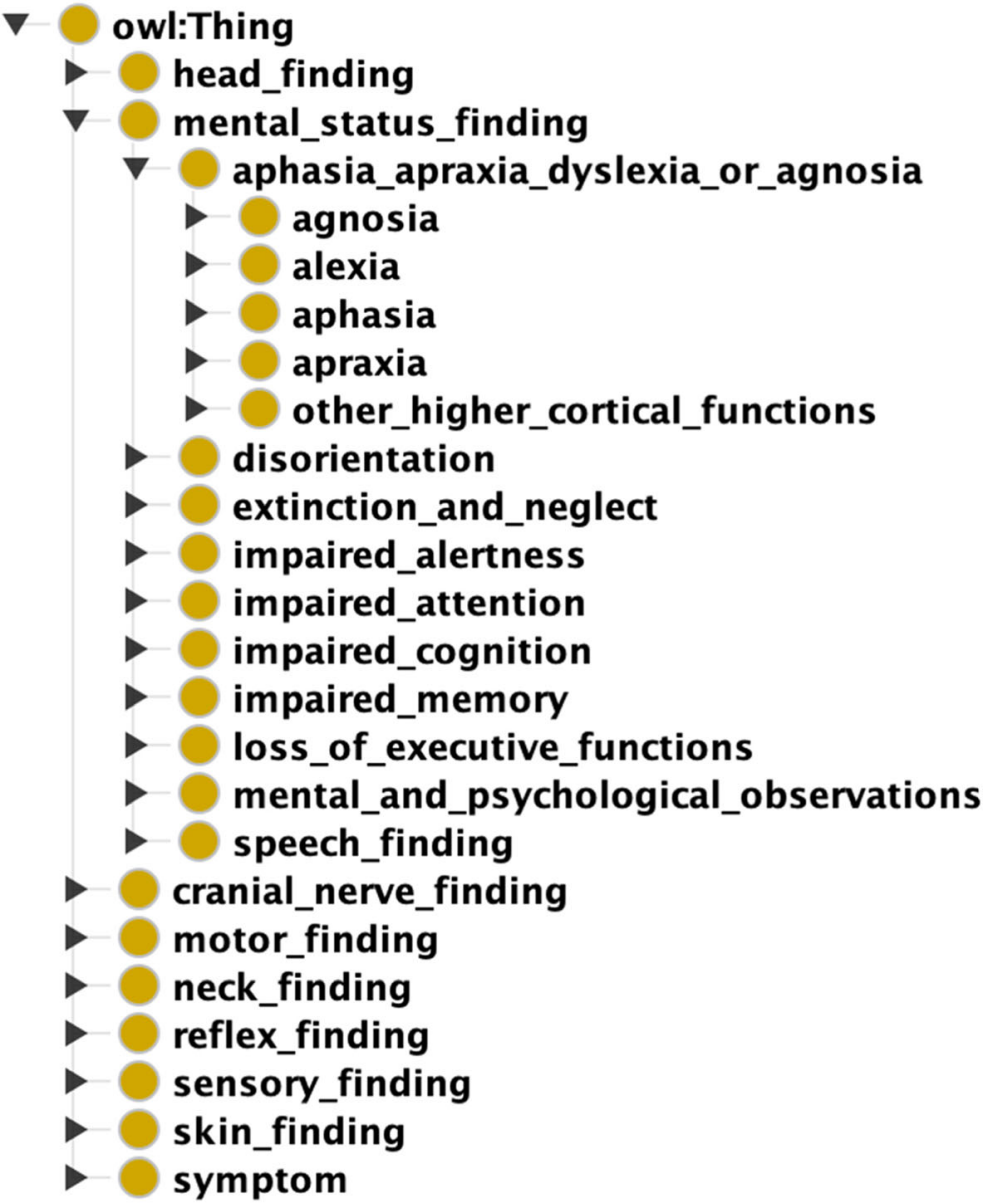
Major branches of NEO as shown in Protégé ontology editor (Image created with Protégé 5.5.0 ontology editor [34]). Mental Status Finding branch is partially expanded further

**Fig. 2:**
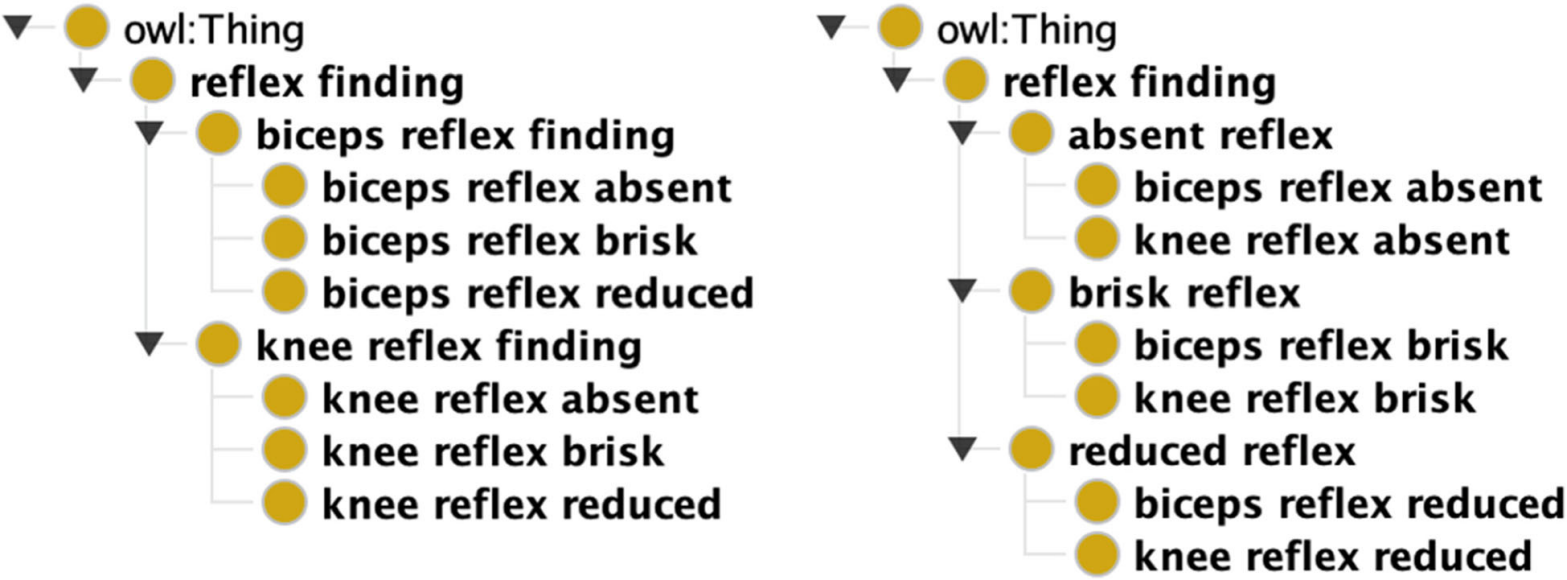
Alternative subsumption strategies for placing brisk biceps reflex in concept hierarchy. Both strategies are semantically correct. Brisk biceps reflex can be grouped with other brisk reflexes (right panel) or with other biceps reflexes (left panel). SNOMED CT groups brisk biceps reflex with bicep reflexes, NEO groups brisk biceps reflex with other brisk reflexes (Image created with Protégé 5.5.0 ontology editor [34].)

We used Protégé to organize the concepts into a mono-hierarchic ontology. The average number of children per concept was 3, and the maximum ontology depth was seven levels. The neuro-ontology is downloadable as a CSV or OWL file at the BioPortal of the National Center for Biomedical Ontology [35]. The neuro-ontology has five high-level branches to mirror the structure of the neurological examination (mental status finding, cranial nerve finding, motor finding, sensory finding, reflex finding) and four additional high-level branches (head finding, neck finding, skin finding, and neurological symptoms). Figure 1 demonstrates a partial expansion of NEO. Table 2 shows the distribution of concepts by the examination section. We had two considerations in building the concept hierarchy for NEO. First, findings related to each part of the neurological examination should be gathered under one consistent heading. Second, the concept hierarchy path distance between findings should reflect a neurologist’s thinking about which concepts are more similar while preserving subsumption. For example, a brisk biceps reflex could be subsumed under either biceps reflex or brisk reflex (see Fig. 2). We chose to subsume brisk biceps reflex under brisk reflex because a brisk biceps reflex is more similar to a brisk knee reflex than it is to an absent biceps reflex.

**Table 2:** Composition Of Target Vocabulary By Major Branch Of Ontology

Category	Count of Concepts
Mental Status	191
Cranial Nerve	222
Motor	211
Sensory	133
Coordination	20
Reflexes	68
Gait and Balance	36
Neck	10
Head	7
Skin findings	6
Symptoms	196
**Total Concepts**	**1100**

### Candidate ontologies for a neurological examination ontology

We identified five component ontologies of the UMLS Metathesaurus (Table 3) as a potential basis for a neurological examination ontology: SNOMED CT, HPO (Human Phenotype Ontology), MEDCIN, MeSH (Medical Subject Headings), and OMIM (Online Mendelian Inheritance in Man). We tested how well each of these ontologies provided concept coverage for the 601 neurology concepts identified above.

**Table 3:** Candidate Ontologies For Use As A Neurological Examination Ontology

	Ontology	Concepts
UMLS	Unified Medical Language System	4,258,810
SNOMED CT	SNOMED CT	357,533
MEDCIN	MEDCIN	348,808
HPO	Human Phenotype Ontology	18,278
OMIM	Online Mendelian Inheritance In Man	109,609
MeSH	Medical Subject Headings	279,425
NEO	Neurological Examination Ontology	1100

### Distance measures between concepts

We calculated distances between concepts for the SNOMED CT and NEO ontologies by the method of Wu and Palmer [24]. Distances between concepts were normalized with a minimum of 0.0 (closest) and a maximum of 1.0 (most distant).

$$ \mathbf{Dist}\ \left(\mathbf{a},\mathbf{b}\right)=1-\frac{2\ast \mathrm{depth}\left(\mathrm{LCS}\right)}{\mathrm{depth}\ \left(\mathrm{a}\right)+\mathrm{depth}\left(\mathrm{b}\right)} $$

**Dist (a, b)** is the semantic distance between concept *a* and concept *b*, ***LCS*** is the lowest common subsumer in the ontology for both a and b, **depth(a)** is number of levels from the root concept to concept a, **depth (b)** is the number of levels from the root concept to concept b, and **depth(LCS)** is the number of levels from the root concept to the LCS.

## Results

We found 2386 neurological examination findings in 419 published neurological teaching cases. On average, each case had 5.7 ± 3.7 neurological findings. Since these cases appeared in neurology textbooks, they likely had more neurological findings per case than can be expected in general neurological practice. These 2386 findings (based on abstracted test-phrases) were mapped to 601 unique UMLS concepts. We examined how well each of the six candidate ontologies was able to cover the 601 test concepts (Table 4). None of the five candidate ontologies had sufficient scope to cover all the concepts. SNOMED CT, the largest of the candidate ontologies, covered 69% of the test concepts. The Neurology Examination Ontology (NEO) constructed from multiple terminologies in UMLS (Table 5), has enough scope to cover all test concepts.

**Table 4:** Coverage Of Test Neurology Concepts By Candidate Ontologies

Ontology	Concepts Covered	Proportion Covered
UMLS Metathesaurus	601	100%
NEO	601	100%
SNOMED CT	412	69%
MEDCIN	317	53%
OMIM	278	46%
HPO	233	39%
MeSH	118	20%

**Table 5:** Terms Contributed To Neurological Examination Ontology (NEO) By Source Vocabulary

Terminology	Concepts	Proportion Contributed
UMLS	1100	100%
SNOMED CT	728	66%
MEDCIN	172	16%
OMIM	76	7%
HPO	52	5%
MeSH	10	1%
Miscellaneous	62	6%

## Discussion

We created a neuro-ontology of 1100 concepts derived from the UMLS Metathesaurus to encode findings from the neurological examination and history with machine-readable codes. To create an ontology with enough scope to cover common neurological findings, we drew concepts from a variety of terminologies (Table 5). We tested the completeness of the Neurological Examination Ontology (NEO) by assessing its ability to encode neurological concepts derived from 419 test teaching cases. These test teaching cases generated 2386 test phrases and 601 unique neurological concepts. NEO had adequate scope to cover 100% of the test concepts with a single concept. SNOMED CT had pre-coordinated concepts to cover 69% of the test concepts (Table 4). Coverage of test neurology concepts by MEDCIN was almost as good as SNOMED CT, while HPO, MeSH, and OMIM lack many key neurological concepts needed to cover the entire neurologic examination and history (Table 4).

Most of the concepts lacking from SNOMED CT were granular concepts needed to describe the motor and sensory examination in detail, such as which muscle groups were weak and the precise distribution and nature of sensory loss (Table 6). In this study, we did not use the post-coordination of concepts to generate more granular concepts. Post-coordination of SNOMED CT concepts would likely have increased the coverage rate. Elkin et al. [36] have shown that post-coordination of concepts can increase coverage for problems on the medical problem list from 50% to over 90%. Post-coordination of concepts is the process of joining together concepts to increase specificity and granularity of meaning. For example, the concept |weakness of right ankle dorsiflexion| can be represented by bringing together the concepts |right| with |ankle dorsiflexion| and |muscle weakness|. UMLS itself does not have a grammar for combining concepts to create post-coordinated concepts. SNOMED CT has a formal compositional grammar that specifies rules for combining concepts (post-coordination). However, the underlying grammar of post-coordination of SNOMED CT concepts is complex and requires considerable training before its successful implementation. Even professional coders may disagree on how to combine concepts to define more complex concepts [37]. Furthermore, calculating semantic distances between post-coordinated concepts and searching databases with post-coordinated concepts is more complicated than with pre-coordinated concepts.

**Table 6:** Examples Of Neurological Concepts Available In UMLS And Not Found in SNOMED CT Browser

CUI	UMLS Term
C2016536	decreased pain and temperature sensation below T2 level
C2054091	tactile sensation decreased sensory level at clavicles (T2 dermatome)
C2039818	decreased tactile sensation of ulnar 1 and ½ digits of hand
C2230515	weakness of ankle on dorsiflexion
C2230516	weakness of ankle plantar flexion
C1847766	shoulder girdle muscle atrophy
C2054045	decreased tactile sensation of lateral leg and dorsum of foot (L5 dermatome)
C2054068	decreased tactile sensation of middle finger only (C7 dermatome)
C2039817	decreased tactile sensation of palmar aspect of radial 3 and ½ digits of hand

For each clinical deficit, the clinical teaching cases used a variety of phrasing to express the same concept. For example, the neurological finding of *bilateral extensor plantar response* was expressed in 13 different ways in the clinical teaching cases (Table 7).

**Table 7:** Test Phrases Mapped To |**C0422917**| Bilateral Extensor Plantar Response

**Test Phrase**	
Babinski response elicited bilaterally	
bilateral Babinski responses	
bilateral Babinski signs	
bilateral extensor toes signs	
bilateral upgoing plantars	
bilateral upgoing toes	
both plantar responses were extensor	
both plantars were upgoing	
both toes upgoing	
plantar responses were extensor	
plantars extensor	
upgoing plantar responses bilaterally	
upgoing plantars	
**UMLS Listed Synonyms**	
Babinski reflexes bilateral	
Bilateral extensor plantar response (finding)	

This heterogeneity of expressions poses challenges for efforts to use natural language processing algorithms to convert free text neurological examinations into UMLS concepts [7, 8]. In a pilot study with NLM MetaMap [38, 39] in the batch mode, we were able to convert 70.3% of the 2286 test phrases to UMLS concepts. A higher conversion yield might be possible with additional post-processing and pre-processing of the longer and more complex test phrases.

### Curation of the hierarchy

**Fig. 3:**
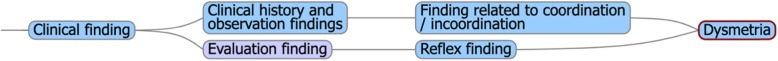
Dysmetria (an imprecision in performing pointing movements with the limbs) is subsumed by incoordination and reflex finding in SNOMED CT. Read hierarchy from right to left (Diagram from Shrimp Ontoserver [42].©Australian e-Health Research Centre)

**Fig. 4:**
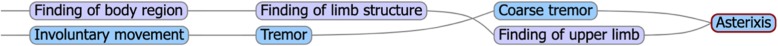
In SNOMED CT, asterixis (a flapping tremor of the arms) is subsumed by coarse tremor and finding of upper limb. Read hierarchy from right to left. (Diagram from Shrimp Ontoserver [42].©Australian e-Health Research Centre)

**Fig. 5:**
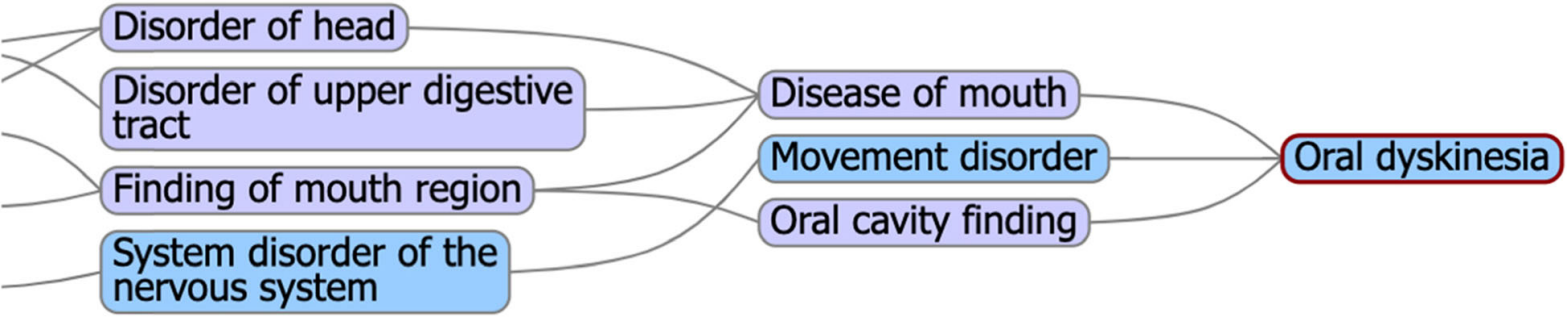
In SNOMED CT, oral dyskinesia is subsumed by disease of mouth, movement disorder, and oral cavity finding. Its defining quality is movement disorder. Read hierarchy from right to left. (Diagram from Shrimp Ontoserver [42].©Australian e-Health Research Centre)

**Fig. 6:**
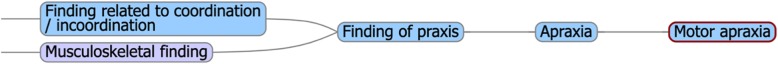
In SNOMED CT concept hierarchy, apraxia is subsumed by finding of praxis and musculoskeletal finding and finding of incoordination. Read hierarchy from right to left. (Diagram from Shrimp Ontoserver [42].©Australian e-Health Research Centre)

One of the advantages of a small domain-specific ontology, such as NEO is that domain experts can more easily identify and correct subsumption errors. Some somewhat arbitrary subsumption decisions may influence how accurately the concept distance measures derived from a concept hierarchy align with expert opinion. Considerable effort has been devoted to finding the best distance metrics for concept hierarchies that give the closest results to expert opinion [19, 20, 22]. However, when the hierarchy itself is not aligned with expert opinion, the choice of distance metric may be less critical. For example, most neurologists would agree that an absent knee jerk is more similar to an absent biceps jerk than it is to a brisk knee jerk (Fig. 2). As a result, in NEO, we have subsumed absent ankle reflex, absent knee reflex, absent biceps reflex, and absent triceps reflex under absent reflex. SNOMED CT subsumes absent ankle reflex and brisk ankle reflex under ankle reflex finding and subsumes absent biceps reflex and brisk biceps reflex under biceps reflex finding. These are ontologically correct (Fig. 2) but yield distance measures that are not in accord with a neurologist’s opinion as to which concepts are more similar (Table 8). In a large ontology like SNOMED CT, some errors in subsumption are inevitable and need to be corrected over time based on input from domain experts [40, 41]. Two examples of likely errors in subsumption are noted in Figs. 3 and 4. Dysmetria is a form of ataxia and hence is correctly subsumed by a finding related to incoordination, but dysmetria is not a reflex finding (Fig. 3). Similarly, apraxia is a disorder of higher cortical functions akin to aphasia and agnosia and should not be subsumed under either incoordination or musculoskeletal disorders (Fig. 4). As a result, distance measures based on the SNOMED CT concept hierarchy show dysmetria as too close to *absent reflexes* and shows *apraxia* too close to *ataxia* (Table 8). One of the goals of SNOMED CT is to have more concepts *fully defined*, which is achieved by adding qualifiers to concepts to make them distinguishable from other concepts in the ontology. Fully defined concepts cannot be confused logically with any other concept in the ontology. However, adding additional subsumption relationships may yield anomalous distance measures between unrelated concepts. In Fig. 5, the subsumption of asterixis (a flapping tremor of the arm) under a finding of the upper limb yields an anomalous distance measure placing asterixis too close to hemiparesis (Table 8). Similarly, in Fig. 6, the subsumption of oral dyskinesia (adventitious spontaneous chewing and writhing movements of the lips and tongue) under oral cavity finding causes oral dyskinesia to be anomalously close to the unrelated concept of vocal cord paralysis (Table 8). Finally, in a small curated domain-specific ontology like NEO, related concepts can be grouped to provide more accurate concept distance measures. For example, by grouping manifestations of bradykinesia such as masked-facies and micrographia close to bradykinesia, distance measures calculated based on the concept hierarchy can reflect the relatedness of these concepts (Table 8).

**Table 8:** Some Examples of Discordant Inter-Concept Distances^a^

First Concept	Second Concept	SNOMED CT Inter-concept distance	NEO Inter-concept distance
brisk ankle reflex	absent ankle reflex	.11	.75
brisk biceps reflex	absent biceps reflex	.17	.75
asterixis	hemiparesis	.23	.64
dysdiadochokinesis	athetosis	.27	.69
dysmetria	absent reflexes	.11	1.00^b^
apraxia	ataxia	.17	1.00^b^
bradykinesia	masked facies	.79	.20
bradykinesia	micrographia	.56	.20
oral dyskinesia	vocal cord paralysis	.33	.67

^a^SNOMED CT and NEO. Distances are normalized with 0.0 as minimum distance, 1.0 as maximum distance. Inter-concept distances were calculated by method of Wu and Palmer based on the concept hierarcy

^b^ When concepts are in different high-order branches of the hierarchy, the distance is 1.0

### Manageability

One advantage of a small ontology like NEO is improved manageability. Both the NLM and SNOMED International recommend the use of subsets of UMLS and SNOMED for certain restricted applications [26, 43]. NEO has only 1100 concepts, and the downloadable CSV file has 1100 rows. In contrast, the UMLS 2019AB release has 4,258,810 concepts, the MRREL.RRF (relationships) file has 84,189,164 rows and the MRCONSO.RRF (concepts) file has 15,172,405 rows. Similarly, the 2019 SNOMED CT US Edition release has 357,533 concepts, the relationship snapshot file has 2,989,896 rows, and the concept snapshot file has 478,117 rows. Since each row in the NEO file has the child-parent relationship for each concept, hierarchies and subsumption tables can be generated directly from the primary file.

### Limitations

A major limitation of this study is that we did not attempt to combine concepts to generate missing or more complex concepts (post-coordination). Because we did not post-coordinate concepts, we were not able to grade muscle weakness when describing the motor examination. Muscle strength is usually graded on a scale of 0 to 5 (0 = ‘no movement’ to 5 = ‘full strength’). We elected to encode the clinical expression |weak quadriceps (4/5)| as |quadriceps weakness| **C0577655** and ignore the degree of weakness. The sensory examination is notoriously difficult to describe in text, and in the era of the paper medical record, neurologists often used drawings to document the sensory examination. For this study, we did not use post-coordinated concepts to describe sensory findings but used instead granular concepts from MEDCIN to describe common sensory findings such as sensory loss in median nerve distribution, sensory loss in C8 dermatome distribution, a sensory level at the T2 level, etc. Similarly, we ignored quantitative visual acuity measurements such as 20/200 and encoded any visual acuity of less than normal as |reduced visual acuity| **C0234632**. Although UMLS has appropriate lateralized concepts for some findings (hemiparesis, rigidity, myoclonus, chorea, tremor, ptosis), there are not separate lateralized concepts for other common neurological findings (ataxia, hyperreflexia, spasticity, hyporeflexia). We did not develop a method to capture these lateralized findings when existing concepts were not available in UMLS and reverted to using the non-lateralized concept (e.g., ataxia for left ataxia).

Another limitation is that we have not validated the phrase abstraction methods or the phrase-to-concept mapping methods with other neurology experts or tested the methodology on de-identified medical records. More work is needed on whether a concise neurological examination ontology such as NEO is useful and acceptable to neurologists.

## Conclusions

With certain limitations, an ontology that is a subset of UMLS with approximately 1100 concepts has adequate breadth and granularity capture the signs and symptoms of the neurological examination and history. Additional concepts may be needed to fully capture the laterality of certain findings (ataxia, hyperreflexia, etc.) as well as the severity of other findings (weakness, spasticity, rigidity, etc.). Using a subset of UMLS concepts to convert neurological signs and symptoms to machine-readable codes offers the advantage of improved manageability and coverage when compared to larger multi-purpose ontologies such as SNOMED CT, MEDCIN, and OMIM.

## Data Availability

This ontology is available for download as a CSV or OWL file at the NCBO BioPortal at http://bioportal.bioontology.org/ontologies/NEO. The test phrases are available on request.

## Abbreviations

CSV: Comma-separated value file
CUI: Concept Unique Identifier
HPO: Human Phenotype Ontology
MEDCIN: MEDCIN® is a registered name of Medicomp Corporation
MeSH: Medical Subject Headings. MeSH® is a registered name of the NLM
NEO: Neurological Examination Ontology
NLM: National Library of Medicine
NLP: Natural Language Processing
OMIM: Online Mendelian Inheritance in Man. OMIM® is a registered name of the McKusick-Nathans Institute of Genetic Medicine
OWL: Web Ontology Language
SCTID: SNOMED CT identifier
SNOMED CT: SNOMED CT® is a registered name of SNOMED International. It is no longer considered an acronym
UMLS: Unified Medical Language System. UMLS® is a registered name of the NLM
